# Effect of Electronic Messaging on Physical Activity Participation among Older Adults

**DOI:** 10.1155/2016/6171028

**Published:** 2016-05-17

**Authors:** Chantrell Antoine Parker, Rebecca Ellis

**Affiliations:** Georgia State University, Atlanta, GA 30302, USA

## Abstract

The purpose of this study was to determine if electronic messaging would increase min of aerobic physical activity (PA) among older adults. Participants were active older adults (*n* = 28; M age = 60 years, SD = 5.99, and range = 51–74 years). Using an incomplete within-subjects crossover design, participants were randomly assigned to begin the 4-week study receiving the treatment condition (a morning and evening text message) or the control condition (an evening text message). Participants self-reported min of completed aerobic PA by cell phone text. The 1-way within-subjects ANOVA showed significant group differences (*p* < 0.05). Specifically, when participants were in the treatment condition, they reported significantly greater average weekly min of aerobic PA (M = 96.88 min, SD = 62.9) compared to when they completed the control condition (M = 71.68 min, SD = 40.98). Electronic messaging delivered via cell phones was effective at increasing min of aerobic PA among older adults.

## 1. Introduction

Regular physical activity (PA) has many physical and mental health benefits for older adults including lowering the risk of early death, improving bone health, increasing cardiorespiratory and muscular fitness, decreasing levels of body fat, and reducing anxiety and depression [1]. To achieve these benefits, the 2008 Physical Activity Guidelines for Americans (PAG) recommend that adults should complete 150 min a week of moderate intensity aerobic PA or 75 min a week of vigorous intensity aerobic PA (or a combination of both), as well as two days a week of muscle strengthening activities [2]. PA participation progressively declines as people age, and currently only 17.1% and 15.9% of adults 55–64 years and 65 years and older, respectively, meet these guidelines; therefore, interventions to increase PA participation among older adults are warranted [3].

Meta-analytic evidence demonstrated that interventions are effective for increasing PA participation [4, 5], and specific characteristics of the intervention produced larger effects on behavior. For instance, in a review of 141 studies, Dishman and Buckworth [5] found significantly larger effect sizes for interventions that used behavior modification strategies such as reinforcement, stimulus control, or behavioral contracts (*r* = 0.92 weighted; *r* = 0.56 not weighted) and for those that were delivered using a mediated approach via indirect implementation through mailings or telecommunications (*r* = 0.91 weighted; *r* = 0.41 not weighted). Behavior modification techniques are based on the premise that the antecedents and consequences (including expected consequences) of the activity influence behavior [6]. Stimulus control involves manipulating environmental conditions, such as using prompts or reminders to decrease the problem behavior (physical inactivity) and increase the targeted behavior (PA participation [6]) and this strategy has frequently been incorporated into health behavior interventions.

In a systematic review of 19 weight loss, PA, and diet interventions that used periodic prompts, Fry and Neff [7] found that 11 studies reported positive intervention effects. The type of prompt (i.e., messages, reminders, and feedback), delivery periodicity (i.e., delivered daily, weekly, or monthly), and method of administration (i.e., sent using e-mail, telephone, and mail) were examined to determine which prompt characteristics had the greatest impact on behavior change. Of the 11 studies with positive results, 5 studies showed significant increases in PA when messages were delivered weekly by telephone and e-mail. Overall, these findings demonstrated that prompts delivered periodically are effective for promoting behavior change, and specifically can increase PA participation. Furthermore, Fry and Neff [7] acknowledge that the use of mobile technology as an alternative method for prompt delivery may be another cost-effective way to promote behavior change that warrants future research.

The use of cell phones, and specifically text messaging, to prompt PA participation is advantageous for promoting healthy behaviors because (a) there is a high penetration of mobile telephones across income and ethnic groups; (b) mobile phones are popular, portable, and convenient; and (c) information can be delivered quickly [8]. Gerber et al. [8] found that participants reported positive feedback and attitudes toward text messaging as a way to cultivate healthy behaviors. In a review of 14 health behavior change interventions that were delivered via mobile telephone short-message service (SMS) text messages, Fjeldsoe et al. [9] observed significant positive behavioral changes in 8 studies, and an additional 5 studies demonstrated positive behavior trends using SMS as a reminder to increase adherence to treatment programs.

Fanning et al. [10] conducted a recent meta-analysis of 11 studies that used mobile devices to increase PA. Specifically, interventions were delivered via SMS (eight studies), mobile software (four studies), and a personal digital assistant (PDA; two studies). The results of the meta-analysis showed that interventions delivered via mobile devices produced significant moderate effects on PA behavior (*g* = 0.54, 95% CI = 0.17 to 0.91, and *p* = 0.01). Moreover, a significant moderate effect was found for those interventions delivered with a mobile phone (*g* = 0.52, 95% CI = 0.11 to 0.94, and *p* = 0.01). However, of the 11 studies reviewed, only 2 reported samples with an average age of at least 60 years [11, 12].

King et al. [11] compared an intervention group that received a programmed alert on their PDA twice a day to a control group that received standard health educational written materials about PA. They found significantly larger increases in PA among older-aged adults when the intervention was delivered via a handheld computer (i.e., PDA). Nguyen et al. [12] compared a mobile self-monitored group to a mobile coached group that sent daily text message updates on exercise and symptoms of COPD. In return, both groups received a weekly thank-you standard text message. However, the mobile self-monitored group did not receive personalized feedback regarding exercise and symptoms of COPD. Although both groups increased PA, the mobile self-monitored group showed significant improvements in total steps per day compared to the mobile coached group. Therefore, using cell phones as a way to merge communication technologies with intervention strategies to increase PA participation in adults warrants more research in general and specifically among older adults.

To date, a limited number of research studies have examined the use of mobile technology to promote PA among adults aged 50 years and older [13–15]. Now that many older adults own and use a mobile phone, the purpose of this study was to determine if electronic prompts delivered via cell phones would increase min of aerobic PA among adults aged 50 years and older. It was hypothesized that participants would report significantly greater average weekly min of aerobic PA during the intervention condition than when they were in the control condition.

## 2. Methods

### 2.1. Participants

Participants were recruited from a certified personal training studio within the Metro-Atlanta area. Study participants met the following inclusion criteria: (1) 50 years of age or older, (2) worked with a personal trainer for at least six consecutive months, (3) currently working with a personal trainer at least twice a week for strength training, but did not meet the PAG for weekly aerobic PA, and (4) able to send and receive e-mail and/or text messages from a cell phone during a 4-week period. Thirty volunteers signed the university IRB approved informed consent form and were given information about the study. Volunteers were between 51 and 74 years of age.

### 2.2. Design and Procedures

This study used an incomplete within-subjects crossover design with counterbalancing of conditions to control for carryover effects between the treatment and control conditions. At the beginning of the 4-week period, the Principal Investigator (PI; the first author) met face to face with each volunteer for about 15 min. During the meeting, the PI explained and received a signature on the informed consent form. Participants also completed the personal history questionnaire. Participants were then randomly assigned to the treatment condition (Group 1) or the control condition (Group 2). During the first two weeks of the study, Group 1 (treatment condition) participants received a morning and evening text message to prompt aerobic PA three days a week. The morning prompt stated “Don't forget to do cardio today” and the evening prompt stated “Did you do your cardio today?” Prompting aerobic PA three days a week was chosen to supplement the two days a week of strength training participants were completing with their personal trainers. Although the participants were not currently meeting the PAG of 150 min a week of aerobic PA, the objective of the research was to test the efficacy of the intervention for increasing weekly min of aerobic PA, not necessarily to achieve 150 min or more. Participants in Group 2 (control condition) received only the evening text message (i.e., “Did you do your cardio today?”) three days a week for two weeks.

All participants completed the electronic PA participation form by cell phone e-mail or text message on the days the text messages were received. Participants were informed that with a “*yes*” response to the evening message, they should report the type of aerobic PA, duration in min, and intensity (moderate or vigorous). With a “*no*” response to the evening message, participants were asked to report the reason for not performing aerobic PA (e.g., barriers). At the end of the first two weeks, participants crossed over to the other condition (i.e., Group 1 completed the control condition and Group 2 completed the treatment condition) and the procedures were executed exactly the same as described during the first two weeks. 

### 2.3. Measures

#### 2.3.1. Personal History Questionnaire

Participants reported age, gender, marital status, race/ethnicity, education, and income. Marital status was categorized as married and other (e.g., domestic partner, single, widowed, and divorced); race/ethnicity was categorized as White and other (e.g., Hispanic or Latino); education was categorized as high school or less, some college or associate's degree, and bachelor's degree or more; and income was categorized as low (<$1306 per month), medium ($1307–$1836 per month), high (≥$25,000 per year), and not reported.

#### 2.3.2. Electronic Physical Activity Participation Form

The electronic PA participation form was used to record the participants' responses to the evening text message during the treatment and control conditions (i.e., “Did you do your cardio today? If yes, what did you do and if no, why not?”). Participants who reported “*yes*” to the completion of aerobic PA provided the type performed (walk, bike, swim, etc.), duration in min, and intensity (moderate or vigorous). Participants who reported “*no*” to the completion of aerobic PA reported barriers that prevented them from engaging in aerobic PA (e.g., no time, work, bad weather, etc.).

### 2.4. Analyses

Tests of outliers and normality were conducted. Participant demographic characteristics were described using frequencies, means, and standard deviations. If outliers were identified and removed from the sample, ANOVA and Chi-square were used to compare group differences between the outliers and remaining sample across demographic variables (e.g., age, gender, marital status, race, education level, and income level). ANOVA and Chi-square were also used to examine group differences across demographic variables between the participants who began the study in Group 1 (treatment condition) and those who began the study in Group 2 (control condition). Self-reported weekly min of aerobic PA and barriers to aerobic PA were summarized using frequencies. Weekly min of aerobic PA were categorized as 0–29 min, 30–59 min, 60–89 min, 90–119 min, 120–149 min, and 150 min or more. Finally, a 1-way within-subjects ANOVA was used to determine significant differences in average weekly min of aerobic PA by condition. SPSS version 18.0 was used to perform all data analyses denoting a statistically significant value of alpha levels at *p* < 0.05.

## 3. Results

Thirty older adults volunteered to participate in the study; however, two were identified as multivariate outliers and removed from the analyses. There were no significant group differences between the outliers and the remaining sample across demographic characteristics except on income level. One of the participants removed as an outlier reported a significantly lower income level (e.g., $1306 or less monthly versus $25,000 or more annually) than the other study participants, *χ* ^2^ (2, *N* = 30) = 14.63, *p* = 0.001; however, it should be noted this was the only volunteer in the sample who reported low income. The final sample included 28 male and female older adults (M age = 60 years, SD = 5.99, Range = 51–74 years; see Table 1). There were no significant group differences between the participants initially randomized into Group 1 (treatment condition) and those who began the study in Group 2 (control condition) across demographic characteristics.

**Figure 1:**
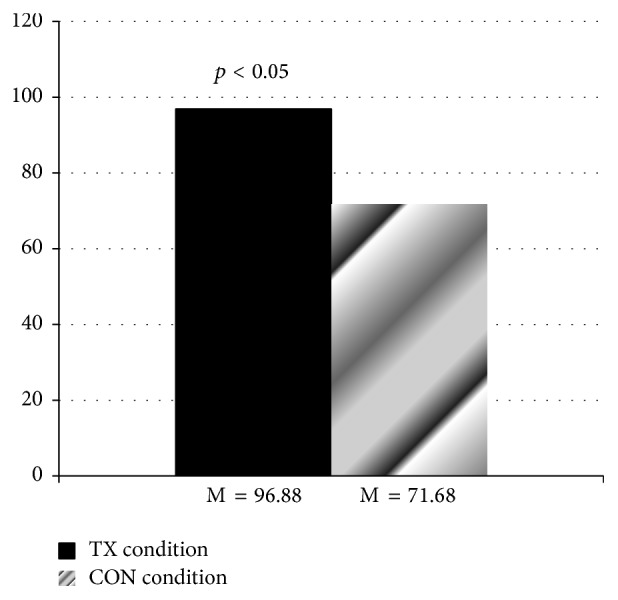
Average weekly min of aerobic physical activity. TX = treatment condition. CON = control condition.

The weekly min of aerobic PA for Group 1 and Group 2 were summarized using frequencies (see Table 2). The 1-way within-subjects ANOVA showed significant differences between conditions on total min of aerobic PA, Wilks' Lambda = 0.82, *F*(1,27) = 5.76, *p* = 0.024, *ηp* ^2^ = 0.18, and observed power = 0.64. Specifically, while participants were in the treatment condition they reported significantly greater average weekly min of aerobic PA (M = 96.88 min, SD = 62.90) compared to when they were in the control condition (M = 71.68 min, SD = 40.98; see Figure 1).

**Figure 2:**
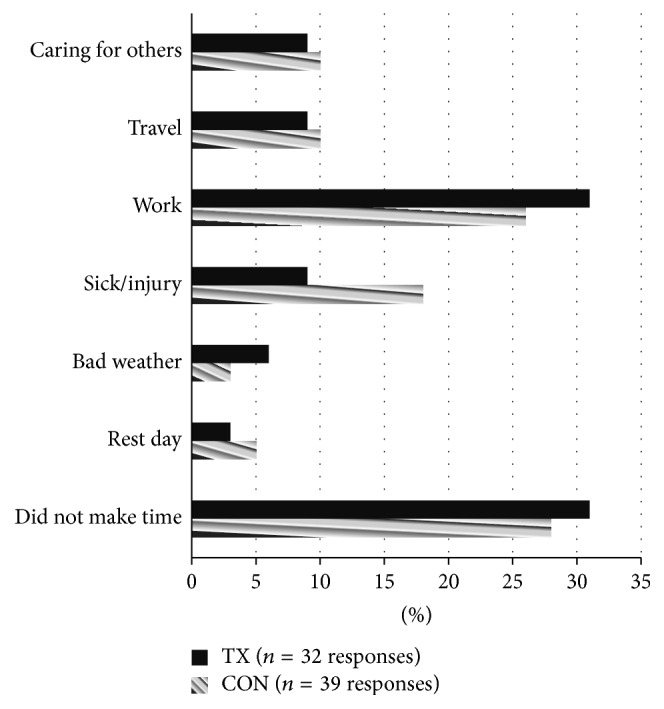
Barriers to aerobic physical activity. TX = treatment condition. CON = control condition.

There were seven common barriers reported among participants that prevented them from engaging in aerobic PA (see Figure 2). The most commonly reported barriers during the treatment condition were (a) did not make time (31%) and (b) work (31%) and the most commonly reported barriers to aerobic PA during the control condition were (a) did not make time (28%), (b) work (26%), and (c) not feeling well (e.g., sick/injury; 18%).

## 4. Discussion

The purpose of this study was to determine if electronic prompts on cell phones would increase aerobic PA participation among adults aged 50 years and older. Average weekly min of aerobic PA were significantly greater during the treatment condition than during the control condition. Although future studies are warranted, current findings are consistent with previous research in that prompts effectively increase PA behavior and demonstrate the promise of using cell phone technology to deliver prompts to older adults. The clinical implications of these findings suggest that this is a feasible and effective intervention strategy for promoting aerobic PA among those 50 years and older who are members and regular users of fitness facilities because this intervention strategy was tested in a real-world setting.

As hypothesized, participants reported significantly greater average weekly min of aerobic PA during the treatment condition when they received the electronic reminder in the morning than during the control condition when the morning reminder was not delivered, and this treatment effect (*p* = 0.024; *ηp* ^2^ = 0.18) was found despite a small sample size and reduced statistical power (0.64). These results are consistent with previous research that demonstrated the effectiveness of prompt interventions with mediated delivery [5, 7–10]. A recent meta-analysis by Fanning et al. [10] found a significant moderate effect for physical activity interventions that were specifically delivered via mobile phones; however, few of the studies reviewed included older adults. Therefore, the findings from this study extend the literature by providing evidence that electronic prompts delivered via cell phones can also be a successful strategy for increasing PA levels among adults aged 50 years and older.

In addition to examining the use of electronic messaging via cell phones to increase PA participation among older adults, common barriers to aerobic PA participation were recorded. The most common barriers during both conditions were “did not make time” and “work,” which are consistent with previous research [16]. However, fewer barriers were reported while participants were in the treatment intervention (*n* = 32) than in the control (*n* = 39), suggesting that the electronic prompts may have assisted with barrier removal. These findings show promise for using electronic prompts delivered via cell phones to increase PA participation as well as to assist with barrier removal.

Although the results of this study suggest that use of electronic prompts delivered via cell phones to promote aerobic PA among older adults is effective, it is not without limitations. First, the generalizability of the findings is limited to a small sample of mostly White, wealthy, well-educated women with access to cell phones who were already physically active (e.g., working with a personal trainer for strength training) and relatively healthy; however, it should be noted that the study sample was representative of the facility population from which volunteers were recruited. Moreover, the study sample was similar to samples included in previous studies that used mobile devices to prompt PA among older adults [11, 12]. These studies also included small samples of mostly White women that held at least a bachelor's degree and earned more than $50,000 a year with an average age of 60 years or more. Second, within a crossover design, although participants were randomized into Group 1 and Group 2, contamination between the groups did occur in participants that partnered together during personal training sessions. Specifically, a husband and wife pair began the study in opposite groups, and the husband in Group 1 asked his wife in Group 2 to walk with him. This contamination may have resulted in increased PA among the control participants.

Finally, the duration of the intervention and the frequency of the electronic prompt delivery may be considered inadequate despite the demonstration of positive results. However, this intervention should be viewed in the context of previous research that shares similar characteristics and also demonstrated the effectiveness of prompts for changing behavior. For instance, interventions that were less than six weeks were included in the Fry and Neff [7] review (see [17, 18]), and the frequency of electronic prompt delivery in this study is within a range of frequencies (e.g., daily, once a week, and once a month) found in studies included in the Fry and Neff [7] and Fjeldsoe et al. [9] reviews (see [11, 12, 17, 19, 20]). In addition, it should be noted that during the treatment condition participants were averaging about 32 min of aerobic PA per bout (6 bouts during 2-week condition) versus 24 min of aerobic PA during the control condition. These values suggest that if the treatment had been delivered 5 times per week, participants were on track to meet the 150 weekly min of aerobic activity. Although longer interventions are necessary to determine the effectiveness of electronic prompts for the maintenance of aerobic PA in older adults, the evidence is promising for the effects of this strategy for promoting adoption and short-term aerobic PA.

## 5. Conclusions

Few studies have examined electronic prompts on cell phones to increase PA participation among older adults [13–15], and few have been tested in real-world settings. The results of this study are consistent with previous research and indicate that electronic prompts can increase aerobic PA among older adults and may assist with barrier removal. Future research interventions using mobile technology are needed to confirm the study findings using a randomized between-subjects design with a larger sample size of older adults across different income levels, educational backgrounds, ethnicities, and health status. Future research interventions should also test the use of the video components (i.e., FaceTime and Skype) of mobile technology for prompt delivery. In summary, the use of electronic prompts on cell phones may be a feasible, cost-effective, and convenient method to increase aerobic PA among older adults. Physicians, physical therapists, and personal trainers may want to consider integrating mobile technology into their practice by using cell phones to deliver reminder, informational, and even instructional prompts to patients and clients.

## Figures and Tables

**Table 1:** Demographics for Group 1 and Group 2 participants.

Characteristic	Group 1		Group 2		Total	
	(*n* = 13)		(*n* = 15)		(*n* = 28)	
	M (SD)		M (SD)		M (SD)	
Age	58.23 (6.59)		61.60 (5.15)		60.04 (5.99)	
Gender						
Male	*n* = 2	15.4%	*n* = 3	20.0%	*n* = 5	17.9%
Female	*n* = 11	84.6%	*n* = 12	80.0%	*n* = 23	82.1%
Marital status						
Married	*n* = 7	53.8%	*n* = 13	86.7%	*n* = 20	71.4%
Other	*n* = 6	46.2%	*n* = 2	13.3%	*n* = 8	28.6%
Race						
White	*n* = 12	92.3%	*n* = 15	100%	*n* = 27	96.4%
Other	*n* = 1	7.7%			*n* = 1	3.6%
Education level						
High school or less	*n* = 2	15.4%	*n* = 1	6.7%	*n* = 1	3.6%
Some college or associate's degree	*n* = 11	84.6%	*n* = 4	26.7%	*n* = 6	21.4%
Bachelor's degree or more			*n* = 10	66.7%	*n* = 21	75.0%
Income level						
$25,000 or more annually	*n* = 12	92.3%	*n* = 10	66.7%	*n* = 22	78.6%
Did not report	*n* = 1	7.7%	*n* = 5	33.3%	*n* = 6	21.4%
History of disease^*∗*^						
Cardiovascular	*n* = 3	23.1%	*n* = 2	13.3%	*n* = 5	17.9%
Cancer	*n* = 0	0.0%	*n* = 3	20.0%	*n* = 3	10.7%
Thyroid	*n* = 0	0.0%	*n* = 3	20.0%	*n* = 3	10.7%
Bone	*n* = 1	7.7%	*n* = 1	6.7%	*n* = 2	7.1%
Spine	*n* = 2	15.4%	*n* = 0	0.0%	*n* = 2	7.1%
Other	*n* = 3	23.1%	*n* = 2	13.3%	*n* = 5	17.9%
No disease reported	*n* = 6	46.2%	*n* = 5	33.3%	*n* = 11	39.3%

*Note.* Group 1 = treatment condition first. Group 2 = control condition first. ^*∗*^Frequencies may not equal total sample sizes or 100% because 3 participants reported 2 illnesses.

**Table 2:** Weekly min of aerobic physical activity for Group 1 and Group 2 participants.

Categories	0–29 min		30–59 min		60–89 min		90–119 min		120–149 min		≥150 min	
Week 1												
Group 1 (TX)	*n* = 2	15.4%	*n* = 3	23.1%	*n* = 1	7.7%	*n* = 1	7.7%	*n* = 4	30.8%	*n* = 2	15.4%
Group 2	*n* = 1	6.7%	*n* = 5	33.3%	*n* = 3	20.0%	*n* = 3	20.0%	*n* = 1	6.7%	*n* = 2	13.3%
Week 2												
Group 1 (TX)	*n* = 2	15.4%	*n* = 2	15.4%	*n* = 2	15.4%	*n* = 2	15.4%	*n* = 1	7.7%	*n* = 4	30.8%
Group 2	*n* = 4	26.7%	*n* = 1	6.7%	*n* = 3	20.0%	*n* = 2	13.3%	*n* = 4	26.7%	*n* = 1	6.7%
Week 3												
Group 1	*n* = 2	15.4%	*n* = 3	23.1%	*n* = 2	15.4%	*n* = 6	46.2%	*n* = 0	0.0%	*n* = 0	0.0%
Group 2 (TX)	*n* = 5	33.3%	*n* = 1	6.7%	*n* = 3	20.0%	*n* = 0	0.0%	*n* = 3	20.0%	*n* = 3	20.0%
Week 4												
Group 1	*n* = 3	23.1%	*n* = 4	30.8%	*n* = 3	23.1%	*n* = 1	7.7%	*n* = 1	7.7%	*n* = 1	7.7%
Group 2 (TX)	*n* = 1	6.7%	*n* = 3	20.0%	*n* = 1	6.7%	*n* = 1	6.7%	*n* = 7	46.7%	*n* = 2	13.3%

*Note.* TX = treatment condition. Group 1 (*n* = 13)** = **treatment condition first. Group 2 (*n* = 15) = control condition first.
